# Engineered biomaterial delivery strategies are used to reduce cardiotoxicity in osteosarcoma

**DOI:** 10.3389/fphar.2023.1284406

**Published:** 2023-10-03

**Authors:** Yulin Hou, Jie Wang, Jianping Wang

**Affiliations:** Department of Cardiology, Guangyuan Central Hospital, Guangyuan, China

**Keywords:** Osteosarcoma, cardiotoxicity, biomaterials, drug delivery, chemotherapy

## Abstract

Osteosarcoma (OS) is the most common malignant bone tumor in children and adolescents. Chemotherapy drugs play an integral role in OS treatment. Preoperative neoadjuvant chemotherapy and postoperative conventional adjuvant chemotherapy improve survival in patients with OS. However, the toxic side effects of chemotherapy drugs are unavoidable. Cardiotoxicity is one of the common side effects of chemotherapy drugs that cannot be ignored. Chemotherapy drugs affect the destruction of mitochondrial autophagy and mitochondria-associated proteins to cause a decrease in cardiac ejection fraction and cardiomyocyte necrosis, which in turn causes heart failure and irreversible cardiomyopathy. Biomaterials play an important role in nanomedicine. Biomaterials act as carriers to deliver chemotherapy drugs precisely around tumor cells and continuously release carriers around the tumor. It not only promotes anti-tumor effects but also reduces the cardiotoxicity of chemotherapy drugs. In this paper, we first introduce the mechanism by which chemotherapy drugs commonly used in OS cause cardiotoxicity. Subsequently, we introduce biomaterials for reducing cardiotoxicity in OS chemotherapy. Finally, we prospect biomaterial delivery strategies to reduce cardiotoxicity in OS.

## 1 Introduction

Osteosarcoma (OS) is the most common malignancy in children and adolescents, accounting for 0.2 percent of all malignancies (Rossi and Del Fattore, 2023). In the US, approximately 4.4 patients per million children and adolescents have OS (Longhi et al., 2006). OS originates from mesenchymal stem cells and occurs in the long diaphyseal epiphysis in children and adolescents, more commonly in the distal femur, proximal tibia, and humerus (Brown et al., 2018; Dong et al., 2022). OS is highly aggressive and often causes pathological fractures and excruciating pain (Franchi, 2012). The prognosis of OS is poor because of its early metastasis and high drug resistance, and the lung is the most common metastatic organ for OS (Farnood et al., 2023). Although surgery and chemotherapy respond well to most malignancies, long-term survival in osteosarcoma is less than 30 percent (Luetke et al., 2014). The rarity of OS and the lack of reliable markers make OS difficult to diagnose and detect early (Beird et al., 2022). The high mortality rate of OS not only brings a heavy financial burden to the patient’s family but also brings great challenges to clinicians.

Surgical resection is usually an effective means of early OS treatment. Amputation is usually the classic treatment for OS, and although it can cure early OS, it can seriously affect the quality of life (Zhang et al., 2022). Neoadjuvant chemotherapy prior to surgical resection and adjuvant chemotherapy after surgery are usually essential (Khadembaschi et al., 2022a). Neoadjuvant chemotherapy reduces the volume of the primary tumor of OS, reduces the rate of OS metastasis, and increases the limb salvage rate of OS (Khadembaschi et al., 2022b). OS without metastasis has a 5-year survival rate of 70 percent (Bielack et al., 2010). Despite new advances in neoadjuvant chemotherapy, effective treatment of OS has not improved (Panez-Toro et al., 2023). This may be related to the fact that OS is resistant to chemotherapy. For chemotherapy-resistant metastatic patients, radiation therapy is another palliative option for prolonging the patient’s life (Ren et al., 2019). Unfortunately, OS cells are not sensitive to radiation therapy, which only prolongs OS survival by 6 months (Ren et al., 2019). Treatment of cases of unresectable metastatic or recurrent OS relies primarily on chemotherapy (Strauss et al., 2021). However, chemotherapy drugs have poor targeting (Panez-Toro et al., 2023). For children and adolescents, timely chemotherapy drugs can target tumor cells, but the side effects of chemotherapy drugs, especially cardiotoxicity, are still a big blow (Heng et al., 2020).

Biomaterials are widely used in various fields of nanomedicine and regenerative medicine because of their biohistocompatibility, targeting, and degradability (Quadros et al., 2021). Over the past few years, a variety of biomaterials have been developed for chemotherapy of malignancies (Xia et al., 2022a). Biomaterials have been developed to encapsulate various chemotherapy drugs and efficiently deliver them to tumor tissue, reducing off-target side effects and side effects of chemotherapy drugs (Zhang et al., 2022). In this article, we first introduce chemotherapy drugs applied to osteosarcoma. Second, we describe the mechanism of cardiotoxicity of chemotherapy drugs. Finally, we summarize and prospect biomaterials for reducing the cardiotoxicity of chemotherapy drugs in the treatment of osteosarcoma.

## 2 Cardiotoxicity of first-line chemotherapy drugs for osteosarcoma

### 2.1 Doxorubicin

Doxorubicin (DOX) is an anthracycline chemotherapy drug used in solid tumors and hematologic malignancies. DOX is widely used and is one of the most commonly used chemotherapy drugs for intermediate and advanced tumors (Chatterjee et al., 2021). DOX plays an important role in cancers such as breast cancer (BC), hematologic tumors, and OS (Rawat et al., 2021). The main mechanism of action of DOX is to inhibit DNA replication and topoisomerase II (Top2) activity of tumor cells, resulting in DNA double-strand breaks that affect the proliferation ability of tumor cells (Corremans et al., 2019). DOX not only has a killing effect on tumor cells, but also has toxic side effects on normal tissue cell fluid, DOX is mainly manifested as nephrotoxicity, hair loss, liver toxicity, chemotherapy brain and bone marrow suppression, and other toxic side effects (Du et al., 2021). But what is more serious about DOX is its toxicity to the heart. Due to its dose-dependent nature, the clinical use of DOX is hampered by life-threatening cardiotoxicity, including cardiac dilation and heart failure (Wu et al., 2023). Studies have shown that DOX induces chronic heart failure beyond its cumulative dose (700 mg/m in adults and 300 mg/m in children) (Xu et al., 2020). Approximately 30 percent of patients develop acute cardiotoxicity following DOX administration, with ST-segment changes, tachycardia, and premature ventricular beats (Ahmad et al., 2022). Most of the symptoms of acute cardiotoxicity can be reversed, and once acute cardiotoxicity persists, it will induce chronic toxicity to the heart. Chronic cardiotoxicity occurs mainly weeks or even months after DOX administration, and the main symptoms are irreversible cardiomyopathy and even congestive heart failure (Koleini et al., 2019). The specific molecular mechanism of DOX cardiotoxicity remains unknown (Feng and Wu, 2023). Studies have shown that the cardiotoxicity of DOX is primarily associated with the destruction of mitochondrial autophagy and mitochondria-associated proteins (Wang et al., 2019). Mitochondrial damage affects mitochondrial substrate metabolism, mitochondrial respiratory chain, and myocardial ATP storage and utilization in tumor cells (Ling et al., 2022). Mechanisms such as oxidative stress, inflammation, iron diesis, imbalanced calcium balance, apoptosis, and autophagy are all implicated in the cardiotoxicity of DOX (Chen et al., 2022).

### 2.2 Platinum-based chemotherapy drugs

Platinum-based chemotherapy is one of the first-line treatments for solid tumors (Weiss and Christian, 1993). The most commonly used platinum chemotherapy drugs in clinical practice are cisplatin, carboplatin and oxaliplatin. Cisplatin is the more commonly used platinum-based chemotherapy agent in the treatment of OS than other platinum-based chemotherapy drugs (Qi et al., 2019). Cisplatin, also known as cis-diamine dichloroplatin, mainly binds to the N7 position on the purine ring and causes DNA damage to tumor cells by blocking cell division leading to apoptosis (Minerva et al., 2023). After DNA damage, tumors lose their ability to proliferate, thereby inducing oxidative stress, upregulating p53, mitogen-activated protein kinase (MAPK) and Jun N-terminal kinase (JNK) or Akt pathways, and inducing apoptosis (Gupta and Nebreda, 2015). Cisplatin also has a killing effect on normal tissue cells, mainly including hepatotoxicity, cardiotoxicity, neurotoxicity, *etc.* (Zhu et al., 2022). Cardiotoxicity with cisplatin alone has been reported to be rare (Jakubowski and Kemeny, 1988). Between 1980 and 2017, only five clinical studies reported cardiotoxicity from cisplatin (Hu et al., 2018). Cisplatin has relatively early cardiotoxic effects, mainly causing arrhythmias leading to ECG changes and chronic heart failure (Fukuhara et al., 2014). Yang et al. reported a decrease in left ventricular ejection fraction (LVEF) from 70% to 48% in a 53-year-old woman with cervical cancer after 3 weeks of cisplatin application (Hu et al., 2018). Although cisplatin alone has not been reported to cause cardiotoxicity, we cannot ignore this problem. The mechanism of cisplatin’s cardiotoxicity remains unknown. Most current views support that cisplatin promotes cardiomyocyte apoptosis by upregulating ROS and mitochondrial DNA disruption leading to mitochondrial dysfunction (Ma et al., 2020).

### 2.3 Methotrexate

Methotrexate is a folate analog that inhibits the activity of dihydrofolate reductase by competing with substrates, resulting in defects in purine and pyrimidine synthesis (Bidaki et al., 2017). Decreased purine and pyrimidine synthesis inhibit tumor cell proliferation, and as a result, methotrexate is also used as an antineoplastic drug (Malaviya, 2016). Methotrexate is mostly used in rheumatoid arthritis and rarely as an antitumor treatment alone (Zhao et al., 2022). Methotrexate is often used in combination with other chemotherapy drugs for antitumor therapy. For example, fluorouracil, doxorubicin, and methotrexate are used for gastric cancer, cyclophosphamide, methotrexate, and 5-fluoropyrimidine are used for advanced breast cancer, and methotrexate, vinblastine, doxorubicin, and cyclophosphamide are used for bladder cancer (Walling, 2006). The chemotherapy regimen in OS is methotrexate-DOX-cisplatin (Smrke et al., 2021). Methotrexate is excreted primarily through the kidneys, and nephrotoxicity is caused by methotrexate crystals in the tubular lumen, leading to tubular toxicity leading to acute kidney injury (Christensen et al., 2012). Methotrexate reduces inflammation, improves cardiovascular risk factors, reduces mortality, and generally has a protective effect on the heart (Bălănescu et al., 2019). However, high doses of methotrexate are toxic to the heart. Shah et al. reported a decrease in ejection fraction and an acute decline in biventricular function in a 54-year-old man with systemic sclerosis after taking methotrexate (Shah et al., 2022). The mechanism of action of methotrexate inducing cardiotoxicity is still very clear. Methotrexate-induced cardiac damage manifests as distortion of normal cardiac tissue structure, significant oxidative and nitrosizing stress, along with decreased glutathione concentration and decreased superoxide dismutase activity, which in turn affects myocardial function (Perez-Verdia et al., 2005; Al-Taher et al., 2020).

### 2.4 Ifosfamide/ifosfamide

Cyclophosphamide is an alkylating agent and immunosuppressant that is widely used in antitumor, organ transplantation, and anti-graft rejection reactions (Song et al., 2016). Cyclophosphamide is primarily used in combination chemotherapy regimens for lymphoma, leukemia, breast, lung, and neuroblastoma (Emadi et al., 2009; Iqubal et al., 2019). Cyclophosphamide binds to guanine residues of tumor cell DNA and causes tumor death (Veal et al., 2016). The pharmacological effects of cyclophosphamide depend on the metabolism of the drug, the dose administered, and the timing of administration (Huyan et al., 2011). The cardiotoxicity of cyclophosphamide is one of the important factors limiting its wide clinical application. Although the mechanism of cyclophosphamide cardiotoxicity has not been completely cleared, the current general consensus is mainly in the following aspects. Cyclophosphamide induces oxygen radical production and inflammation in cardiomyocytes, and its metabolites also reduce the production of endothelial nitric oxide synthase phosphorylation (Iqubal et al., 2019). Cyclophosphamide also induces activation of the p53 and p38 mitogen-activating protein kinase pathways, leading to cardiac apoptosis, inflammation, and hypertrophy (DeJarnett et al., 2014).

Ifosfamide is more commonly used in chemotherapy for OS than cyclophosphamide (Spalato and Italiano, 2021). Similar to cyclophosphamide, ifosfamide is a cell-cycle, nonspecific antineoplastic agent that can be hydrolyzed by phosphoramidase to phosphamide mustard in humans for antitumor effects (Palmerini et al., 2020). The most common adverse effect of ifosfamide is central system toxicity, which occurs in nearly 20 percent of patients with severe hallucinations, confusion, or episodes of drowsiness and coma called ifosfamide encephalopathy (Lee Brink et al., 2020; Ilyas et al., 2021). The toxic effects of ifosfamide on the heart are also not fully understood. Similar to cyclophosphamide, ifosfamide induces oxygen radical production and inflammation in cardiomyocytes, inducing apoptosis in cardiomyocytes (Savani and Skubitz, 2019). Chemotherapy regimens of doxorubicin, cisplatin, ifosfamide, and/or high-dose methotrexate are considered first-line chemotherapy agents for OS (Marec-Berard et al., 2020). However, the cardiotoxicity of chemotherapy drugs is inevitable, and the development of biomaterials to deliver chemotherapy drugs to reduce cardiotoxicity is the development trend of OS therapy.

## 3 Biomaterials reduce cardiotoxicity of OS chemotherapy

### 3.1 Extracellular vesicles

Extracellular vesicles (EVs) are vesicle-like structures with a diameter of 30–150 nm produced by living cells (Lee and Kim, 2021). As an emerging mode of intercellular communication, EVs can deliver RNA, proteins, and other carriers to target cells, thereby regulating the proliferation and differentiation of target cells and affecting the structure and function of target cells (Chang et al., 2021). The advantage of EVs as delivery vehicles is that they can fuse membranes with target cells without destroying the carrier five, thereby releasing the carrier into the target cell (Xia et al., 2022b). EVs produced by cells from different sources have cell-homing effects at different locations in the body (Wiklander et al., 2015). This property makes EVs uniquely targeted. Chemical modifications also give EVs more precise targeting ability, and EVs have more systemic toxic side effects than other biological materials. Wei et al. used bone marrow-derived EVs to deliver DOX (EVs-Dox) to reduce the cardiotoxicity of DOX in OS therapy (Wei et al., 2022). The EVs loaded with DOX have a diameter of 178.1 nm. EVs-Dox is more toxic to tumor cells than DOX and much less toxic to cardiomyocytes than DOX. EVs-DOX can target OS cells *in vivo*, effectively inhibiting the proliferation and migration of OS cells. At 12 h *in vivo*, the concentration of EVs-DOX in cardiomyocytes was much lower than in the DOX group. This is mainly due to the fact that Evs can express SDF-1 protein, and the interaction of Stromal cell-derived factor-1 (SDF-1) and C-X-C motif chemokine receptor 4 (CXCR4) induces DOX-loaded EVs to tend to osteosarcoma sites, reducing the accumulation of cardiomyocytes and reducing the cardiotoxicity of DOX.

### 3.2 Liposomes

**FIGURE 1:**
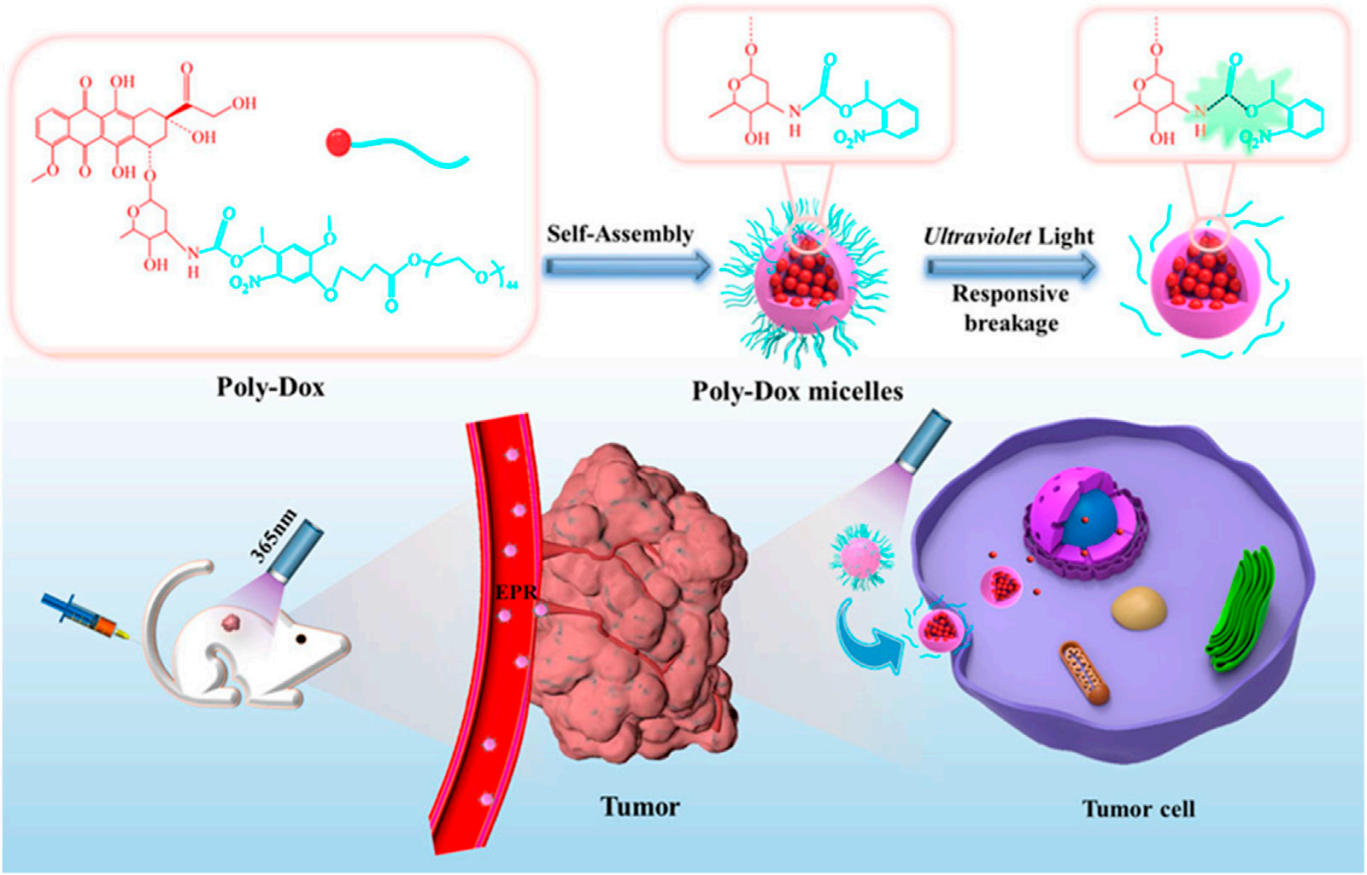
Schematic illustration of the self-assembly and responsive breakage of Poly-Dox micelles, and the process of tumor therapy. DOX self-assembles into photoresponsive polymeric micelles. After tail vein injection in mice, polymeric micelles can aggregate around tumor cells due to the EPR effect. Increase the concentration of DOX in tumor cells to achieve anti-tumor effect. Reproduced with permission from (Chen et al., 2021).

The FDA-approved DOX liposomal preparation, Caelyx, is significantly less cardiotoxic and well tolerated (De Sanctis et al., 2015). DOX liposomes reduce the expression of P-glycoprotein, which is the main mediator of DOX cardiotoxicity. OS also expresses CD44 receptors. Gazzano et al. made HA-Lsdox by coupling DOX liposomes with hyaluronic acid (HA) (Gazzano et al., 2019). HA is a donor to CD44, and the high expression of CD44 in OS makes it an ideal target to increase HA-Lsdox delivery to tumors. HA-Lsdox has a size range of 190 nm–204 nm and effectively releases DOX *in vivo* to promote apoptosis of tumor cells. HA-Lsdox can also promote sulfation and ubiquitination of P-glycoprotein, reducing the cardiotoxic effect of DOX. Nanomicelles can enhance the enhanced permeability and retention (EPR) effect of tumor cells, and the inert groups on the surface of nanomicelles can effectively reduce the uptake of drugs by other normal tissue cells, thereby effectively reducing the side effects of drugs (Riley et al., 2019). Chen et al. synthesized a photoresponsive DOX conjugated polymer (Poly-Dox-M) (Figure 1) (Chen et al., 2021). Nanomicelles indicate cross-linked polyethylene glycol (PEG), which is stable *in vivo* after self-assembly into Poly-Dox-M due to the inert nature of PEG. Poly-Dox-M has a diameter of 27 nm and a drug loading rate of 13.62%. The surface-inert PEG is decomposed by amide bond destruction after several minutes of UV irradiation, promoting the release of DOX by Poly-Dox-M. *In vivo*, experiments have shown that Poly-Dox-M accumulates in large numbers of tumor cells compared to DOX, while the heart, spleen, liver, lungs, and kidneys are very small. As a result, Poly-Dox-M reduces the toxic side effects caused by DOX. This may be due to the lack of ultraviolet light exposure to vital organs, and Poly-Dox-M cannot break down the DOX that releases its load, thus reducing the visceral toxicity of DOX. The cardiotoxic effects of DOX liposomes in reducing DOX have been clinically proven. Huang et al. demonstrated that ifosfamide combined with DOX liposomes for OS treatment can reduce cardiotoxicity compared with ifosfamide plus DOX (Huang et al., 2022). Yang et al. demonstrated that PEG-DOX clinically reduces the cardiotoxicity of DOX (Yang et al., 2020).

### 3.3 Nanoparticles

**FIGURE 2:**
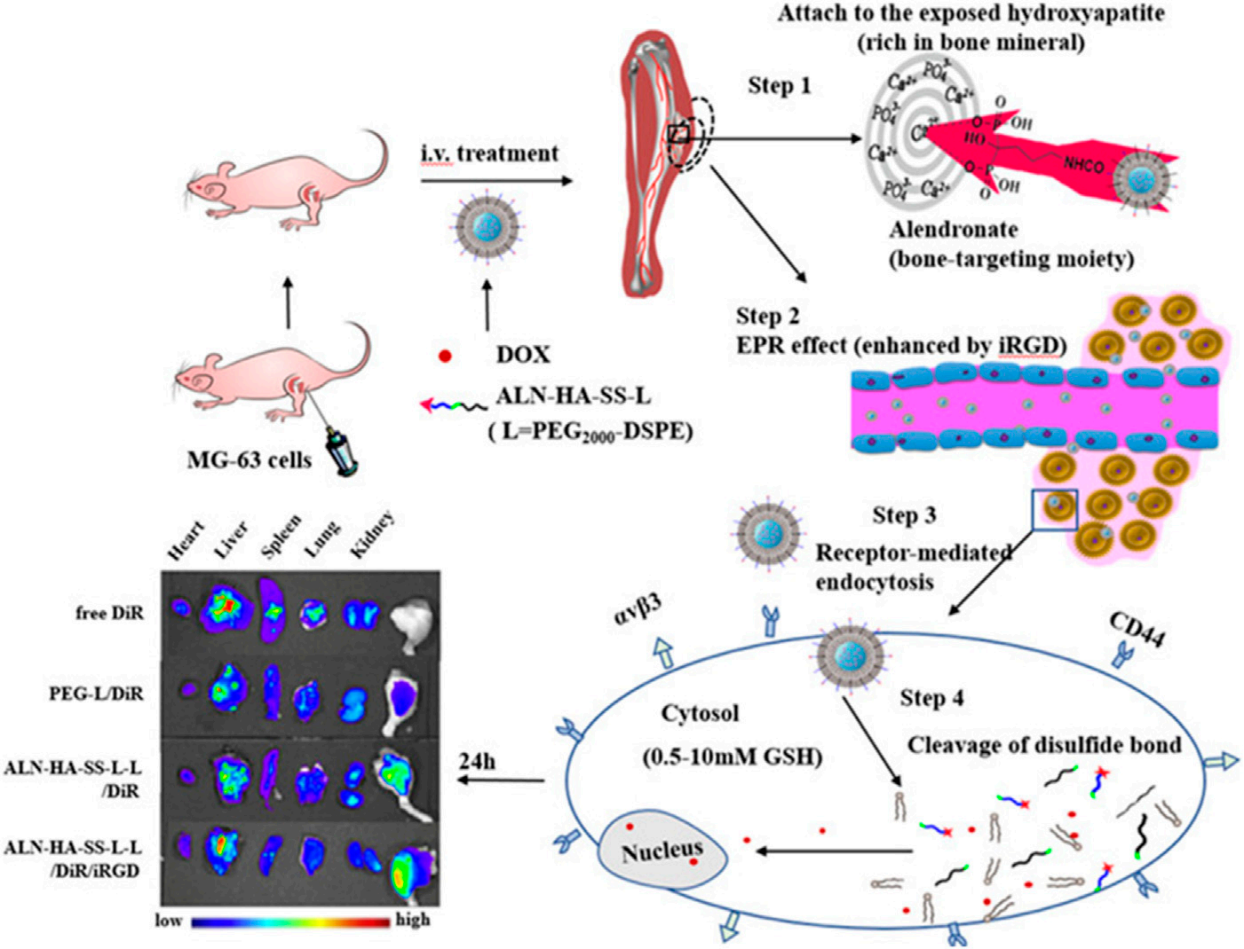
Alendronate (ALN) is coupled to the CD44 ligand hyaluronic acid (HA). The ALN-HA conjugate was conjugated to DSPE-PEG 2000–COOH to obtain functionalized lipid ALN-HA-SS-L by a bioreducible disulfide bonder (−SS−), which was inserted into a preformed liposome loaded with doxorubicin (DOX). ALN-HA-SS-L-L/DOX is significantly more cytotoxic to human OS MG-63 cells with high and rapid cell uptake. The antitumor effects of various liposomes are the same as those shown by *in vivo*/*ex vivo* imaging. In the *in situ* OS nude mouse model, ALN-HA-SS-L-L/DOX showed significant tumor growth inhibition and prolonged survival. Reproduced with permission from (Feng et al., 2019).

Wang et al. made PCP-PEG-ALD nanoparticles from aldehyde rubicin (ALD), positively charged proteins (PCP), and PEG (Wang et al., 2021). The solubility of ALD is extremely low, and poor bioavailability limits its clinical use (Gong et al., 2018). PCP-PEG-ALD nanoparticles have good biocompatibility. PCP-PEG-ALD nanoparticles have a diameter of 200 nm, which has a hihg encapsulation rate for ALD and can deliver ALDto tumor cells. PCP-PEG-ALD nanoparticles can kill tumor cells (survival rate 29%) without causing damage to normal cells (survival rate 95%). Compared with DOX-induced focal necrosis of cardiomyocytes, PCP-PEG-ALD nanoparticles only cause mild symptoms of myocardial stromal adiposia. Feng et al. used the principle that redox-sensitive nanoparticles containing disulfide bonds were used to respond to the redox potential of tumor cells, and coupled bone-targeted partial alendronate (ALN) with the CD44 ligand hyaluronic acid (HA) to form functionalized liposomes (ALN-HA-SS-L-L) for DOX delivery (Figure 2) (Feng et al., 2019). ALN-HA-SS-L-L is able to target bone tumors *in vivo* and break disulfide bonds within tumor cells to release DOX to achieve anti-tumor effects. ALN-HA-SS-L-L released 3% and 72% of DOX in the first 6 h and 23 h, respectively. *In vivo* experiments, free DOX treatment was confirmed to cause severe cardiotoxicity and liver and kidney damage, while ALN-HA-SS-L-L/DOX treatment reduced cardiotoxicity and inhibited lung metastases.

### 3.4 Hydrogels

Hydrogels are plastic, adherent, biocompatible, and biodegradable and widely used in tissue engineering and nanomedicine (Liang et al., 2023). Topical delivery systems for hydrogel drugs are able to deliver anticancer drugs directly to the target, reducing the toxic effects of chemotherapy drugs (Yoo et al., 2018). β-cyclodextrin (β-CD) can be used as a nanoscale drug carrier for anticancer drugs, enhancing the water solubility of drugs (Li et al., 2015). Sun et al. made DOX, cisplatin-loaded β-CD into nanoscale HP-β-CD drug-delivery hydrogels for combination chemotherapy for OS (Yoon et al., 2019). HP-β-CD hydrogels continuously release DOX and cisplatin for anti-tumor effects. *In vitro* experiments have confirmed that HP-β-CD hydrogel can reduce the proliferation rate of tumor cells. HP-β-CD hydrogel can reduce tumor volume *in vivo* for 4 weeks. Cao et al. used a hydrogel-microsphere (Gel-Mps) complex composed of collagenase (Col) and PLGA microspheres (Mps) to carry pioglitazone (Pio) and Dox to achieve combined chemotherapy for OS (Cao et al., 2023). Gel-MPS has a highly biodegradable, extremely efficient, and low-toxicity sustained drug release effect, showing an effective inhibitory effect on tumor proliferation. The average size of Mps is 3.97μm, and the load ratios of Dox and Pio are 3.79% and 13.69%, respectively. *In vitro* experiments, Gel-Mps was able to achieve sustained release of Dox and Pio within 20 days. *In vivo* experiments have shown that Gel-Mps can reduce the migration and proliferation of tumor cells. The Pio released by Gel-Mps can also reduce the expression of P-glycoprotein, and reduce cardiotoxicity and resistance to chemotherapy drugs.

### 3.5 Polymer brackets

**FIGURE 3:**
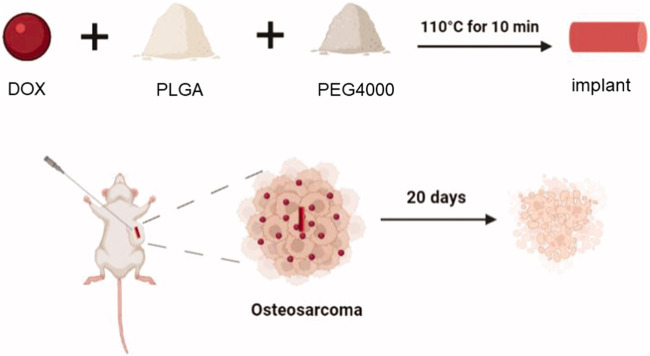
The schematic illustration of the DOX-loaded implant for intratumoral chemotherapy. Preparation of the DOX-loaded implant. DOX, PLGA, PEG are made of degradable polymer scaffolds, and polymer scaffolds can continuously release DOX into the tumor to achieve anti-tumor effects. After 20 days, tumor cells were observed to shrink. Reproduced with permission from (He et al., 2022).

OS often leads to increased destruction, often resulting in bone non-regeneration and severe pain (Whelan and Davis, 2018). Bioscaffolds can provide support around OS, providing a foundation for bone regeneration. He used poly(-lactide-collective) and polyethylene glycol (PEG) to make bioscaffolds for loading DOX (Figure 3) (He et al., 2022). The average diameter of the biological scaffold was (0.86 ± 0.03) mm and the length was (4.22 ± 0.26) mm, and the loading ratio of DOX was 78.0% ± 6.32%. *In vitro* experiments showed that the polymeric scaffold released about 26.4% of DOX within 2 hours, and was able to release all DOX within 15 days. Compared with DOX, DOX in stents can achieve *in situ* release around the tumor, reduce DOX accumulation in the heart, and reduce cardiotoxicity. Polymethyl methacrylate (PMMA) is a bone substitute with excellent formability, high mechanical strength, and appropriate biocompatibility (van Vugt et al., 2019). PMMA can release heat, causing thermal necrosis of tumor cells, resulting in local antitumor effects (Gaston et al., 2011). However, the thermal effects of PMMA often cause normal cell necrosis, and cement leakage also limits the widespread use of PMMA cement (Dolan et al., 2012). Wang et al. used carboxymethylcellulose (CMC) to increase the porosity of PMMA cement for enhanced cisplatin delivery (Wang et al., 2022). The addition of CMC reduces the compressive strength of PMMA. Bone cement has a porosity of 30–40 μm and a cisplatin loading rate of 16.6% ± 1.1%. Bone cement continuously releases cisplatin within 14 days to achieve the chemotherapy effect of bone cement.

## 4 Conclusion and outlook

As one of the most important organs of the human body, the role of the heart cannot be ignored. Although the cardiotoxicity of chemotherapy drugs is not fully understood, it has been agreed that they affect the ejection fraction of the heart and cardiomyocyte function, and thus cause heart failure and cardiomyocyte death by inhibiting mitochondrial activity. Although chemotherapy drugs have achieved good results in the treatment of advanced tumors, the adverse effects of chemotherapy drugs are often unbearable and painful for patients. Chemotherapy drugs kill tumor cells as well as normal tissue cells. This is a puzzle that clinicians cannot solve. How to reduce the toxicity of chemotherapy drugs remains a difficult problem for clinicians. The emergence of biomaterials has made great strides in tissue engineering and nanomedicine. Biomaterial delivery strategies have made major breakthroughs in bone regeneration, anti-tumor, tissue repair, and regenerative medicine. Nanoparticles, hydrogels, scaffolds, *etc.*, based on PLGA, PEG, chitosan, gelatin, alginate, bioactive glass, *etc.*, have been designed for anti-tumor treatment, and their effects have been confirmed in preclinical studies. Although biomaterial delivery strategies have achieved good results in preclinical studies, few have been truly applied clinically. At present, albumin paclitaxel chemotherapy drugs are widely used in clinical practice as a mature chemotherapy drug. However, other nanomaterials for delivery are still only in their initial stages. Preclinical research still needs to address the biological activity of biomaterials *in vivo* meet the persistence of antitumor therapy and reduce the side effects of chemotherapy drugs. Future research still needs to further develop biomaterials to reduce the cardiotoxicity of chemotherapy drugs. Based on the results achieved in current preclinical studies, we believe that sooner or later the toxic effects of biomaterials in reducing the toxicity of chemotherapy drugs will be applied to the clinic on a large scale.
